# Clinical Research Analysis and Application Value of the Laparoscopic Retroperitoneal Approach in In Situ Splenectomy

**DOI:** 10.1155/grp/7113085

**Published:** 2026-09-18

**Authors:** Dong-Fang Huang, Jian-Bo Xu, Ye-Mu Du, Ye-Bo Wang

**Affiliations:** ^1^ Department of Hepatobiliary Surgery, The Affiliated Huaian No. 1 People′s Hospital of Nanjing Medical University, Huaian, China, njmu.edu.cn

**Keywords:** laparoscope, posterior approach, splenectomy

## Abstract

**Objective:**

The purpose of this study was to compare the effects of the laparoscopic posterior approach in in situ splenectomy with those of traditional splenectomy. The primary indications for splenectomy in our cohort were splenomegaly and hypersplenism secondary to liver cirrhosis and portal hypertension, which is a common clinical scenario in our hepatobiliary surgery‐centred hospital.

**Methods:**

A retrospective analysis was conducted on 58 patients who underwent splenectomy in our hospital. These were divided into 28 cases of laparoscopic posterior approach in situ splenectomy (posterior approach group) and 30 cases of traditional open splenectomy (open splenectomy group). Surgical indicators and postoperative complications were compared between the two groups.

**Results:**

Intraoperative blood loss, length of hospital stay and time to drainage clearance were substantially lower in the posterior approach group compared with the open splenectomy group. There was no significant difference in operation time. Postoperative complications in the posterior approach group included fever (7 cases), ascites (14 cases), pancreatic leakage (0 cases), rebleeding (1 case), secondary abdominal infection (0 cases) and venous thrombosis (2 cases). These were fewer than in the open splenectomy group, which presented with 18 cases of fever, 24 of ascites, 7 of pancreatic leakage, 4 of rebleeding, 3 of secondary abdominal infection and 6 of venous thrombosis. Statistically significant differences were observed in the incidence of pancreatic leakage, fever and ascites between the groups (*p* = 0.011, *p* = 0.007 and *p* = 0.016, respectively). No significant differences were found for rebleeding (*p* = 0.392), secondary abdominal infection (*p* = 0.238) or venous thrombosis (*p* = 0.299).

**Conclusion:**

The laparoscopic retroperitoneal approach in in situ splenectomy demonstrates clear advantages over traditional splenectomy with respect to intraoperative performance and postoperative complications, offering improved safety and efficacy.

## 1. Introduction

Traditional splenectomy, also known as open splenectomy, is a surgical procedure to remove the spleen through open surgery [1]. The disadvantage is that the recovery time is longer, the length of hospital stay is relatively long and the incidence of postoperative complications is relatively high, including infection, rebleeding and spleen functional problems. Postoperative pain is more intense, and activity during the recovery period is limited [2–4].

To solve these problems, laparoscopic splenectomy (LS) was introduced [5]. LS is a minimally invasive surgery which has been widely used in clinical situations such as splenomegaly, splenic rupture, splenic tumours, autoimmune diseases (such as idiopathic thrombocytopenic purpura), splenic parasite infection, haematological diseases and hypersplenism [6–8]. In this study, the majority of indications were for hypersplenism and splenomegaly due to cirrhosis and portal hypertension, reflecting the patient population commonly treated in our hepatobiliary surgery specialty centre. Compared with traditional open surgery, this operation has the advantages of less trauma, faster recovery and shorter hospitalisation time. It has become an important option for modern surgical treatment of spleen diseases, which can effectively improve patients′ quality of life and health status [9].

Although LS has become relatively mature, different surgical approaches have their own advantages and disadvantages. The traditional lateral approach is commonly used in splenectomy. Doctors are experienced and skilled in this approach, which can shorten the operation time and provide good anatomical vision and ease of operation. However, this method may increase the risk of injury to the ribs and intercostal nerves, aggravate postoperative pain, interfere with surrounding tissues and increase the risk of pulmonary complications and spinal compression [10]. Compared with the posterior approach, the lateral approach involves a relatively larger incision and longer recovery time [11, 12].

Relatively speaking, LS via the posterior approach has attracted increasing attention in recent years. This approach provides a broader view of the spleen from the back, which supports more effective identification and handling of splenic vessels [13]. Compared with the lateral approach, it has less impact on the intercostal nerves, and postoperative pain is generally milder. Clear visual access allows surgeons to operate with greater accuracy and minimises damage to surrounding blood vessels, thereby reducing the risk of bleeding. The minimal trauma involved in this procedure also promotes quicker recovery, leading to shorter hospital stays [14].

Therefore, the purpose of this study is to evaluate the clinical application of total laparoscopic posterior approach in situ splenectomy and compare it with traditional splenectomy. The incidence of postoperative complications, such as pancreatic leakage, rebleeding, abdominal infection, venous thrombosis and ascites, has been analysed in both the posterior approach group and the open splenectomy group to assess the safety of the posterior approach. Operation time, intraoperative blood loss, length of hospital stay and drainage clearance time have also been compared to comprehensively evaluate the advantages and clinical applicability of this technique.

## 2. Study Objectives and Methods

### 2.1. Study Participants

This study retrospectively analysed the clinical data of 58 patients who underwent splenectomy in our hospital between March 2023 and December 2023. After informing the benefits and risks, the patients chose the surgical method on their own. However, the final decision was made after comprehensive evaluation by the surgeon, considering factors such as spleen size, severity of cirrhosis and presence of extensive perisplenic adhesions, to ensure the safety and feasibility of the laparoscopic procedure. According to the surgical method used, the patients were divided into 28 cases of laparoscopic posterior in situ splenectomy (posterior group) and 30 cases of traditional splenectomy (open splenectomy group).

The inclusion criteria included the following: (1) Patients had a clear diagnosis of spleen diseases such as splenomegaly, splenic tumours or idiopathic thrombocytopenic purpura (the primary indication being hypersplenism secondary to liver cirrhosis and portal hypertension); (2) patients met the clinical indications for LS, and the surgeon had fully evaluated the necessity of surgery; (3) patients were aged between 18 and 75 years; (4) patients were in good general condition, with preoperative evaluation consistent with the relevant anaesthesia and surgical requirements for the few patients with Child–Pugh Grade C cirrhosis (*n* = 5 across both groups); surgery was considered only after meticulous multidisciplinary assessment and optimisation, aiming to control severe hypersplenism and thrombocytopenia in selected individuals where conservative management had failed and (5) patients agreed to participate in the study and signed informed consent.

The exclusion criteria included the following: (1) patients with severe heart and lung disease, liver or kidney dysfunction or other systemic conditions affecting surgical safety, (2) patients with active infection or severe immunosuppression, (3) patients with malignant tumours with metastasis beyond the spleen, (4) patients with abnormal spleen anatomy or other surgical contraindications, (5) pregnant or lactating women and (6) patients with severe mental disorders or cognitive impairment who could not understand the study content or provide informed consent.

### 2.2. Study Methods

#### 2.2.1. Preoperative Preparation

Before surgery, 58 patients received transfusions of concentrated platelets (10 units). Each patient received an intravenous infusion of cefotaxime antibiotics 30 min before surgery and continued to use antibiotics for 4–5 days after the procedure. Routine blood examinations were performed on Days 1, 3 and 7 after the operation. When the platelet count exceeded 400 × 10^9^/L, oral aspirin was given for antiplatelet aggregation, or intravenous infusion of low molecular dextran was administered. The amylase level in the abdominal drainage fluid was measured on the 3rd day after surgery to evaluate postoperative recovery.

#### 2.2.2. Surgical Method of Laparoscopic Posterior Approach in In Situ Splenectomy

All patients underwent surgery under general anaesthesia with tracheal intubation, taking a supine position with the legs separated in a herringbone shape. The pneumoperitoneum pressure was maintained at 12–14 mmHg (1 mmHg = 0.133 kPa). The laparoscopic instruments used were from Karl Storz GmbH & Co. KG (Tuttlingen, Germany), including a 30‐degree laparoscope, ultrasonic scalpel (HARMONIC ACE+), and Endo GIA Universal Staplers (Medtronic, Dublin, Ireland). The placement of surgical instruments and trocars was as follows: (1) The first trocar (10 mm) was located below the navel and served as the lens entry, (2) the second trocar (12 mm) was placed 2 cm below the costal margin of the left clavicle and used as the main operating port, (3) the third trocar (5 mm) was located 2 cm below the xiphoid and also served as a main operating port, (4) the fourth trocar (12 mm) was positioned at the left anterior axillary line as the auxiliary operating port and (5) the fifth trocar (5 mm) was located 2 cm below the costal margin of the anterior axillary line and used as a secondary operating port. Sufficient distance was maintained between each pair of trocars to ensure that the instruments did not interfere with each other inside the abdominal cavity. Except for the umbilical port, the positions of the other ports were adjusted vertically according to the size of the spleen. (Please refer to the revised Figure S1 for a clearer illustration of the port placement.)

**Figure 1:**
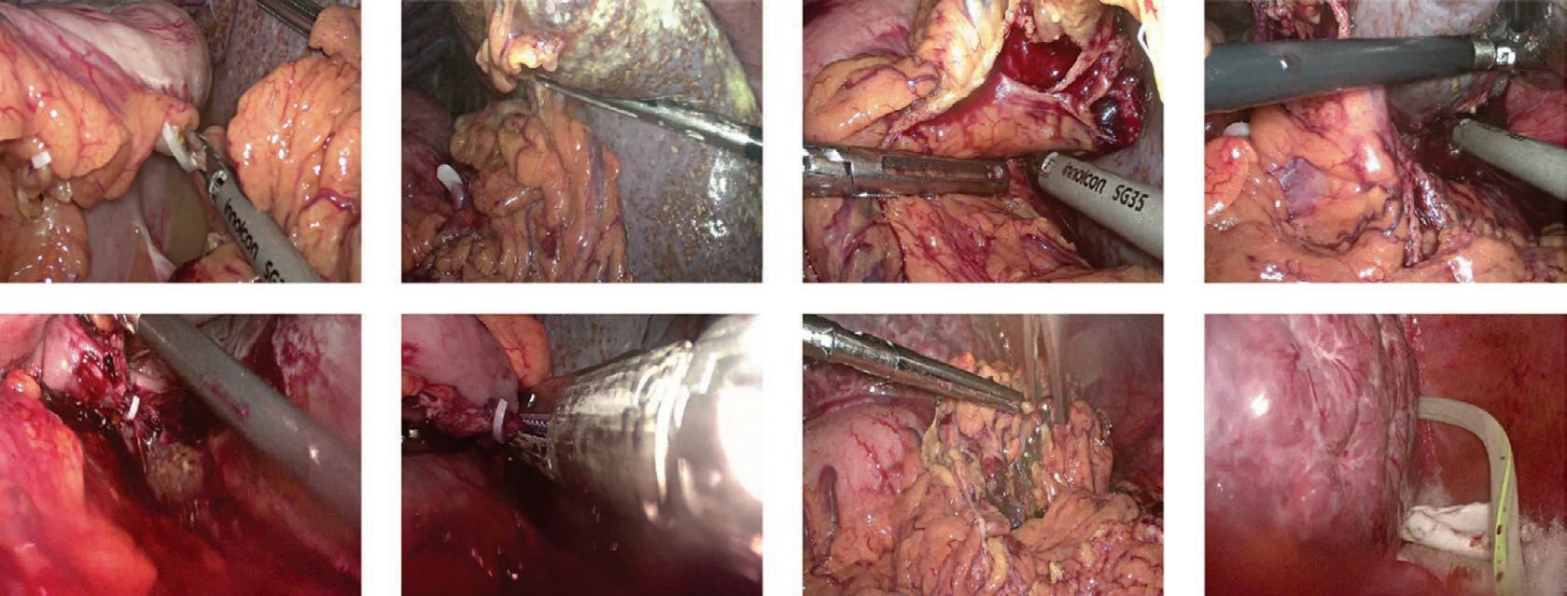
Specific surgical operation diagram. Open the gastrocolic ligament and expose the upper pole of the spleen. Ligating and cutting off the splenic artery, the volume of the spleen began to shrink; the colour darkened. The operating forceps were passed through the gap between the spleen and kidney ligaments to the lower pole of the spleen to establish a tunnel. The splenic hilum vessels and ligaments were severed with the Endo GIA cutting closure device, and the splenic colon ligaments and some lower splenic vessels were severed with an ultrasonic scalpel. The short gastric vessels were cut off with an ultrasonic scalpel. Ultrasound separation of the splenic diaphragmatic ligament. The stump of the pancreatic tail and the wound of the Endo GIA cutting closure device were clipped with titanium clips. The drainage tube was routinely placed in the splenic fossa.

The operation steps were as follows (Figure 1): (1) Abdominal exploration was performed to check for ascites, accessory spleens and tumours in the abdominal and pelvic cavities and to evaluate the degree of cirrhosis and adhesion; (2) the ligament was cut along the middle of the gastrocolic ligament using an ultrasonic scalpel to expose the upper part of the spleen; (3) the splenic artery sheath was opened at the root or middle part of the splenic artery, and the splenic artery was clipped using a Hem‐o‐lok ligation clip to reduce the volume and darken the colour of the spleen. Subsequently, part of the gastrosplenic ligament was opened, the short gastric vessels were cut, the splenic pedicle tunnel was established and the splenic portal vessels and ligaments were cut using the Endo GIA cutting closure device; (4) the left gastroepiploic vessels were cut along the gastrocolic ligament to expose the lower pole of the spleen, and the splenocolic ligament and some lower pole vessels of the spleen were cut using the ultrasonic scalpel; (5) the patient was placed in the head‐high, right lateral position to expose the short gastric vessels, which were disconnected using the ultrasonic scalpel and clamped with Hem‐o‐lok and titanium clips; (6) the lower pole of the spleen was lifted, the splenorenal ligament was gradually cut and the splenophrenic ligament was separated to manage adhesions. Finally, bipolar electrocoagulation was used to stop bleeding at the stump of the pancreatic tail, and the resected spleen was placed in a collection bag and removed through the left upper abdominal incision; (7) a drainage tube was placed in the splenic fossa, and the abdominal incisions were sutured to complete the operation.

For the open splenectomy group, a standard procedure was performed. Under general anaesthesia, a left subcostal incision or midline laparotomy was made. The gastrocolic ligament was divided to access the lesser sac. The splenic artery was identified, ligated and divided. The short gastric vessels were ligated and divided. The spleen was mobilised by dividing the splenorenal, splenophrenic and splenocolic ligaments. The splenic hilum was carefully dissected, the vessels were ligated en masse or individually and the spleen was removed. A drain was placed in the splenic fossa before closure.

### 2.3. Evaluating Indicators

The operation time, intraoperative blood loss, length of hospital stay, drainage clearance time and complications were recorded and compared between the two groups. Postoperative complications included fever (defined as body temperature > 38.5°C occurring after the first 24 h postoperatively), ascites, pancreatic leakage, rebleeding (defined as postoperative haemorrhage requiring surgical reintervention), secondary abdominal infection and venous thrombosis (venous thrombosis referred specifically to splenic or portal vein thrombosis, diagnosed by Doppler ultrasound or computed tomography). The criteria for drain removal were a daily output of less than 50 mL with nonbilious and nonsanguinous character.

### 2.4. Statistical Analysis

Statistical analysis was performed using SPSS 19.0 statistical software. The Kolmogorov–Smirnov test was used to assess the normality of quantitative data. Data conforming to a normal distribution were described using the $\text{mean}\pm \text{standard}\;\text{deviation}\;\left(\overset{¯}{x}\pm s\right)$. An independent samples *t*‐test was used to compare the means between the two groups. Count data were expressed as frequency (*n*) or rate (%). The *χ* ^2^ test was used when applicable, and Fisher′s exact test was used when the conditions for the *χ* ^2^ test were not met. A *p* value of < 0.05 was considered statistically significant.

## 3. Results

### 3.1. General Data

There were 17 men and 11 women in the posterior approach group, with an average age of 46.11 ± 3.79 years. In terms of liver function grading, there were 16 cases of Child Grade A, 10 cases of Grade B and 2 cases of Grade C. In the open splenectomy group, there were 17 men and 13 women, with an average age of 46.20 ± 2.73 years. According to the Child classification, there were 17 cases of Grade A, 10 cases of Grade B and 3 cases of Grade C. There was no significant difference in gender, age or liver function grade between the two groups (*p* > 0.05). There was also no significant difference in preoperative general data between the two groups (*p* > 0.05), as shown in Table 1.

**Table 1:** Comparison of general data of patients.

Variable	Posterior approach group (*n* = 28)	Open splenectomy (*n* = 30)	*t*/*χ* ^2^value	*p* value
Mean age	46.11 ± 3.79	46.20 ± 2.73	−0.107	0.915
Gender			0.098	0.754
Male	17	17		
Female	11	13		
Child grade			—	1.000^a^
A	16	17		
B	10	10		
C	2	3		
Surgical indications			1.205	0.547^a^
Hypersplenism	18	20		
Varicose bleeding	7	6		
Symptomatic splenomegaly	3	4		
Liver cirrhosis portal hypertension	14	16	0.001	0.901
Preoperative platelet count	71.35 ± 47.84	73.64 ± 49.73	−0.754	0.344
Preoperative spleen size (cm)	21.56 ± 4.85	21.84 ± 4.78	0.322	0.749
Preoperative serum haemoglobin	112.35 ± 24.87	109.87 ± 21.71	1.862	0.125
INR	1.4 ± 0.3	1.5 ± 0.4	0.751	0.415
APTT (s)	38.2 ± 5.1	39.6 ± 6.2	0.574	0.289
Preoperative ascites grading				
No ascites	18	15	0.753	0.301
Small (< 500 mL)	7	9	0.823	0.612
Medium (500–1000 mL)	3	5	0.469	0.478
Large (> 1000 mL)	0	1	0.743	0.481
Spleen weight (g)	620 ± 150	650 ± 180	0.763	0.521

^a^Fisher′s exact probability method is used.

### 3.2. Comparison of Surgery‐Related Indicators Between the Two Groups of Patients

In terms of surgery‐related indicators, intraoperative blood loss (220.29 ± 43.87 mL), hospital stay (8.39 ± 1.29 days) and drainage time (3.21 ± 0.92 days) in the posterior approach group were significantly lower than those in the open splenectomy group (intraoperative blood loss: 269.40 ± 23.77 mL; hospital stay: 11.47 ± 1.89 days; drainage time: 4.93 ± 0.91 days). The differences were statistically significant (*p* < 0.001). There was no significant difference in operation time between the two groups, as shown in Table 2.

**Table 2:** Comparison of operation time, intraoperative blood loss, hospitalisation time and drainage clear time between the two groups of patients.

Group	Number	Operation time (min)	Intraoperative blood loss (mL)	Length of stay (days)	Drainage clear time (days)
Posterior approach group	28	134.79 ± 17.57	220.29 ± 43.87	8.39 ± 1.29	3.21 ± 0.92
Open splenectomy	30	139.83 ± 17.07	269.40 ± 23.77	11.47 ± 1.89	4.93 ± 0.91
*t* value		−1.110	−5.350	−7.192	−7.173
*p* value		0.272	< 0.001	< 0.001	< 0.001

### 3.3. The Incidence of Postoperative Complications in the Two Groups of Patients

In terms of postoperative complications, the number of cases in the posterior approach group was as follows: fever (7 cases), ascites (14 cases), pancreatic leakage (0 cases), rebleeding (1 case), secondary abdominal infection (0 cases) and venous thrombosis (2 cases). These were all lower than the corresponding numbers in the open splenectomy group: fever (18 cases), ascites (24 cases), pancreatic leakage (7 cases), rebleeding (4 cases), secondary abdominal infection (3 cases) and venous thrombosis (6 cases). The differences in the incidence of fever and ascites were statistically significant (*p* = 0.007 and *p* = 0.016), and the incidence of pancreatic leakage was also significantly lower in the posterior approach group (*p* = 0.011). Other complications, including rebleeding (*p* = 0.392), secondary abdominal infection (*p* = 0.238) and venous thrombosis (*p* = 0.299), showed no significant difference between the two groups, as shown in Table 3.

**Table 3:** Comparison of postoperative complications between the two groups.

Group	Number	Fever	Ascites	Pancreatic leakage	Rebleeding	Secondary abdominal infection	Venous thrombus
Posterior approach group	28	7	14	0	1	0	2
Open splenectomy	30	18	24	7	4	3	6
*χ* ^2^ value		7.234	5.769	—	0.732	—	1.077
*p* value		0.007	0.016	0.011^a^	0.392	0.238^a^	0.299

^a^Fisher′s exact probability method is used.

## 4. Discussion

With the continuous development of laparoscopic technology, the clinical application of posterior splenectomy is expected to become a new standard for the treatment of splenic diseases, providing more choices for surgeons. This is of great significance for improving the quality of life of postoperative patients [15]. Studying the advantages of the posterior approach provides a scientific basis for determining indications, selecting surgical methods and evaluating prognosis in splenectomy. This helps reduce the incidence of surgery‐related complications and improves patient safety [16, 17].

Unlike traditional LS, a key feature of the posterior splenectomy is that the perisplenic ligaments do not need to be severed initially. Instead, a tunnel is first established for the splenic hilum, through which the hilum is ligated and the splenic vessels are severed. Only then are the perisplenic ligaments around the spleen released.

This study can also provide data to support improvements and innovations in related surgical techniques, thereby promoting the use of minimally invasive surgery in other organs and fields. By discussing the effects and mechanisms of different approaches, it enhances the theoretical understanding of LS and offers a reference for further research.

In summary, this study not only provides evidence‐based recommendations for surgical approach selection in clinical practice but also introduces a new perspective for research and development in the surgical field.

This study compared operation‐related indicators between the posterior approach group and the open splenectomy group. The results showed that the posterior approach group had substantially lower intraoperative blood loss, hospitalisation time and drainage time than the open splenectomy group. These findings have important clinical value.

The intraoperative blood loss in the posterior approach group was 220.29 ± 43.87 mL, which was significantly lower than 269.40 ± 23.77 mL in the open splenectomy group (*p* < 0.001). This may be related to the anatomical characteristics of posterior approach surgery. The posterior approach effectively reduces the risk of bleeding by minimising direct traction and damage to blood vessels. In addition, the use of finer anatomical techniques and less tissue damage during surgery may make the treatment of blood vessels more appropriate, further reducing the occurrence of intraoperative bleeding. This result supports the view that the posterior approach technique is safer and more effective in the implementation of splenectomy.

The hospitalisation time of the posterior approach group was 8.39 ± 1.29 days, which was significantly shorter than 11.47 ± 1.89 days in the open splenectomy group (*p* < 0.001). The shorter hospital stay is closely related to the minimally invasive nature of the surgical technique and the faster postoperative recovery. After undergoing posterior approach surgery, patients can usually get out of bed earlier and resume daily life more quickly due to the lower incidence of postoperative complications and reduced pain. This not only improves the quality of life for patients but also reduces the use of hospital resources, which reduced healthcare costs due to shorter hospitalisation.

The drainage clearance time in the posterior approach group was 3.21 ± 0.92 days, also significantly shorter than 4.93 ± 0.91 days in the open splenectomy group (*p* < 0.001). The shorter drainage time is generally associated with fewer negative effects and may be due to less intra‐abdominal tissue damage and a lower risk of postoperative fluid accumulation. Posterior approach surgery helps accelerate drainage clearance by reducing abdominal injury and promoting faster elimination of lymph and other body fluids.

Although the posterior approach group showed substantial advantages in other indicators, there was no significant difference in operation time between the two groups. This may be because the complexity of the surgery and the required technical skills are not substantially affected by the change in surgical approach. However, it also suggests that clinical practitioners should consider multiple factors, such as surgical efficiency and postoperative recovery, when selecting the surgical method.

The results regarding postoperative complications also showed that the incidence of complications in the posterior approach in situ splenectomy group was considerably lower than that in the traditional oblique approach group. Specifically, the incidence of postoperative fever, ascites and pancreatic leakage showed statistically significant differences, indicating that the posterior approach may offer an advantage in reducing postoperative adverse events. In traditional open surgery, to fully expose the hilum of the spleen, it is often necessary to extensively dissect the perisplenic ligaments (such as the splenorenal and splenophrenic ligaments) and manipulate the tail of the pancreas. This can increase the risk of mechanical injury to the pancreatic duct.

Postoperative fever is usually a sign of infection or an inflammatory response. The posterior approach may reduce the risk of postoperative infection by minimising damage to surrounding tissues, thereby decreasing the likelihood of fever [18]. The formation of ascites is often related to lymphatic leakage or intra‐abdominal inflammatory reactions. The posterior approach may help reduce the incidence of ascites by limiting intra‐abdominal trauma and injury to lymphatic vessels [19]. Pancreatic leakage is one of the more serious complications following splenectomy and is typically associated with pancreatic injury [20].

The posterior approach establishes a splenic hilum tunnel through a natural anatomical space (such as the posterior space of the spleen and kidney ligament) to avoid direct traction on the tail of the pancreas. In traditional open surgery, due to limitations in the surgical field and operative angle, it may be necessary to excessively mobilise the pancreatic tail to expose the splenic hilar vessels, which increases the risk of mechanical damage to the pancreatic parenchyma or pancreatic duct. The posterior approach technique substantially reduces the likelihood of accidental injury to the pancreatic tail by following a precise retroperitoneal anatomical path.

In this study, the incidence of pancreatic leakage was 0% in the posterior approach group and 23.3% in the open splenectomy group. This result is consistent with the conclusion reported by Wang et al. [17], indicating that the posterior approach can reduce pancreatic complications. Rebleeding is usually associated with postoperative vascular coagulation insufficiency. The posterior approach helps reduce the risk of postoperative bleeding through more accurate anatomical dissection and less tissue damage.

In addition, after laparoscopic surgery, the patient′s limited mobility may lead to an increased risk of venous thrombosis, but posterior approach surgery may contribute to earlier activity due to rapid recovery, thereby reducing the incidence of venous thrombosis.

Although the posterior approach technique shows advantages in many aspects, the study also points out that its equipment demand is high and the cost is relatively high. The use of special instruments (such as cutting closures and electrocoagulators) may lead to a substantial increase in patient medical expenses. This limitation has been emphasised in some literature, and researchers call for more in‐depth discussions across different industry settings and levels of patient affordability.

This study investigated the clinical outcomes of laparoscopic in situ splenectomy via the posterior approach; however, several limitations should be acknowledged. These include a relatively small sample size, short follow‐up duration, the nonrandomised nature of the study design and the use of single‐centre retrospective data. Although strict inclusion criteria (e.g. regarding age, liver function and surgical indications) were applied to minimise heterogeneity, the potential for selection bias remains. The assignment to surgical approach was determined by patient preference combined with the surgeon′s clinical judgement rather than randomisation. As a result, patients with more complex conditions—such as significantly enlarged spleens or severe cirrhosis—may have been disproportionately assigned to the open surgery group, which could influence the comparability of complication rates between the two approaches.

Therefore, future studies should expand the sample size and conduct multicentre randomised controlled trials to improve the reliability and external validity of the results and increase long‐term follow‐up to comprehensively evaluate postoperative complications and patients′ quality of life. In addition, the development of innovative surgical techniques and technologies, economic evaluation and scientific clinical pathways will help further improve the safety and efficacy of surgery. By addressing these challenges, future research will promote the advancement of this minimally invasive surgical technique and provide better treatment options for more patients.

## 5. Conclusion

This study showed that laparoscopic in situ splenectomy was superior to traditional splenectomy in terms of intraoperative blood loss, hospital stay and postoperative complication rate, particularly in the incidence of pancreatic leakage, rebleeding and secondary abdominal infection. These results indicate that this surgical method has good safety and effectiveness, and it is recommended for wider application in clinical practice. Further research is needed to verify these findings and to optimise surgical strategies.

## Funding

No funding was received for this manuscript.

## Conflicts of Interest

The authors declare no conflicts of interest.

## Supporting information


**Supporting Information** Additional supporting information can be found online in the Supporting Information section. Figure S1: Distribution map of port.

## Data Availability

The data that support the findings of this study are available from the corresponding author upon reasonable request.
