# Supplementary material for: Clinical Research Analysis and Application Value of the Laparoscopic Retroperitoneal Approach in In Situ Splenectomy
Source: Gastroenterol Res Pract. 2026 Sep 18;2026:7113085. doi: 10.1155/grp/7113085 (PMC13586995; doi:10.1155/grp/7113085)
Supplement: Supplementary file 1 [file GRP-2026-7113085-s001.doc]

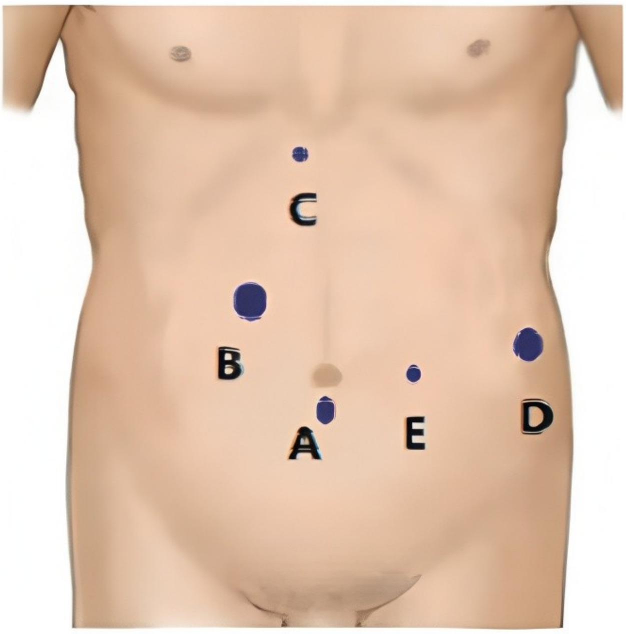

A: Under the navel level for the lens hole.
B: A 12 mm puncture device was placed horizontally above the right umbilicus of the right clavicle midline as the main operating hole.
C: A 5 mm puncture device was placed under the xiphoid for the main operation hole.
D: A 12 mm perforation hole was inserted into the left anterior axillary line as the auxiliary operating hole.
E: A 5 mm puncture device was placed horizontally at the lower edge of the spleen of the left clavicle midline as a secondary operating hole.
